# Functional Analysis of Carotenoid Biosynthetic Genes and Pathway of the Filamentous Anoxygenic Phototrophic Bacterium *Chloroflexus aurantiacus*

**DOI:** 10.3390/genes17101189

**Published:** 2026-09-25

**Authors:** Jiro Harada, Ken Yamamoto, Shinichi Takaichi

**Affiliations:** 1 Department of Medical Biochemistry, Kurume University School of Medicine, Fukuoka 830-0011, Japan; yamamoto_ken@med.kurume-u.ac.jp; 2 Department of Molecular Microbiology, Tokyo University of Agriculture, Sakuragaoka, Setagaya, Tokyo 156-8502, Japan; shintakaichi@gmail.com

**Keywords:** carotenogenesis, carotenoid, *Chloroflexota*, chlorosome, filamentous anoxygenic phototrophic bacteria, lycopene, oxidative stress, photosynthetic bacteria, phytoene desaturase, β-carotene ketolase, CrtO, CrtI

## Abstract

**Background/Objectives:** Carotenoids play essential roles in light harvesting, photoprotection, and oxidative stress defense in photosynthetic organisms. The filamentous anoxygenic phototroph *Chloroflexus aurantiacus* synthesizes diverse carotenoids; however, the biosynthetic basis and regulation of these pigments are unclear. **Methods:** Carotenoid compositions of *Cfx. aurantiacus* grown under various conditions were analyzed by HPLC. Furthermore, candidate carotenoid biosynthesis genes from *Cfx. aurantiacus* were heterologously expressed in *Escherichia coli* to analyze their functions. **Results:** We examined carotenoid composition in cells grown under anaerobic light, aerobic dark, and aerobic light conditions, and found that the major carotenoid species changed markedly in response to oxygen availability. Under anaerobic photosynthetic conditions, γ-carotene, β-carotene, and hydroxylated γ-carotene derivatives were predominant, whereas aerobic growth induced the accumulation of keto-carotenoids, including canthaxanthin and echinenone-related compounds. Genome mining revealed candidate carotenoid biosynthesis genes, including three *crtI* paralogs and genes encoding enzymes involved in cyclization, hydroxylation, glucosylation, esterification, and ketolation. Heterologous expression in *E. coli* revealed that CrtI1 acts as a phytoene desaturase, CrtY catalyzes cyclization of lycopene to β-carotene via γ-carotene, CruF functions as a 1,2-hydratase, CruC as a carotenoid glucosyltransferase, CruD as an acyltransferase involved in esterification, and CrtO as a β-carotene ketolase. **Conclusions:** These results establish a carotenoid biosynthetic pathway in *Cfx. aurantiacus* and show that oxygen-dependent transcriptional regulation, particularly of CrtO, contributes to the selective production of keto-carotenoids under aerobic conditions. Our findings provide new insight into carotenoid metabolism in filamentous anoxygenic phototrophs and the adaptive physiological strategies that support growth under contrasting environmental conditions.

## 1. Introduction

Carotenoids are isoprenoid pigments found in a wide range of organisms. They are particularly important to photosynthetic organisms. Because of the conjugated double bonds (Figure 1) in their linear backbone, they have an excellent capacity to absorb visible light energy, which dissipated as heat [1,2,3]. Carotenoids in the triplet state can quench triplet chlorophyll and singlet oxygen, a reactive oxygen species [4,5,6]. This allows carotenoids to function as photoprotective pigments. In photosynthetic organisms, carotenoids are located in antenna systems and photosynthetic reaction centers, where they perform these functions.

Green sulfur bacteria (GSBs) contain an antenna system known as the chlorosome, which has the highest energy-harvesting capacity among known photosynthetic systems [7]. Chlorosomes consist of many self-assembled bacteriochlorophyll (BChl) *c*, *d*, or *e* molecules enveloped in a lipid monolayer comprising specific proteins and a BChl *a*-containing CsmA protein complex called the baseplate [8]. In addition, approximately 10% (mol/mol) of carotenes per BChl are present in the chlorosomes [9]. The reaction center primarily contains OH-chlorobactene glucoside ester [10]. Energy absorbed by chlorosomes is transferred to Type-I reaction centers [11,12]. In the chlorosomes and reaction centers, carotenoids are present. The GSB *Chlorobaculum* (*Cba.*) *tepidum* primarily synthesizes chlorobactene [13,14], which is a carotenoid unique to GSBs (Figure S1). Studies of carotenoids in GSBs have advanced considerably through mutant analyses of *Cba. tepidum*. Carotenoids contribute to (1) accessory light harvesting at wavelengths outside the absorption spectrum of the BChl self-assembly within the chlorosome, (2) stabilization of reaction center structure [15,16], and (3) prevention of photo-oxidative damage in the presence of oxygen [17]. The precise mechanisms remain unclear, and carotenoids are thought to function by quenching the triplet state of BChl *a* in the CsmA baseplate protein at the base of the chlorosome.

Carotenoids have mainly C40 hydrocarbon chains, eight isoprenoids, and conjugated double bonds. Their biosynthetic pathway begins with phytoene, which is synthesized from farnesyl pyrophosphate by geranylgeranyl pyrophosphate (GGPP) synthase (CrtE) and phytoene synthase (CrtB) [18,19,20,21,22] (see Figure 1). Lycopene is synthesized by desaturation of four double bonds in phytoene. There are two types of enzymes for this process. One is the bacterial-type phytoene desaturase (CrtI), which synthesizes lycopene through a four-step desaturation of phytoene, and is studied in purple bacteria and non-photosynthetic bacteria (see Figure 1). Some purple bacteria possess a three-step desaturation CrtI, which produces neurosporene [22,23,24]. The other is the plant-type enzyme observed in GSBs, cyanobacteria, and plants, and involves the two-step phytoene desaturase CrtP and the two-step ζ-carotene desaturase CrtQ to synthesize lycopene, together with the carotene isomerase CrtH [13,18,20,25,26] (see Figure S1). Most modifications of carotenoids are started from lycopene. Lycopene is cyclized at both ends to β-carotene via γ-carotene. Three types of lycopene cyclases have been reported: CrtY/CrtL, CrtYc/CrtYd, and CruA/CruP [13,21,22,27,28,29]. The CrtY/CrtL-type is widely distributed in bacteria including phototrophs and aerobic non-phototrophic bacteria, and chloroplasts in algae and land plants. The CruA/CruP-type is found only in GSBs and cyanobacteria. Analysis of a gene-deletion mutant of *Cba. tepidum* revealed that CruA is functional. The CrtYc/CrtYd-type is found in some prokaryotes, archaea, and fungi. Mutagenesis studies in *Cba. tepidum* have also revealed several novel genes encoding an isorenieratene synthase (CrtU), a carotenoid glucosyltransferase (CruC), a glucoside esterase (CruD), and a 1,2-carotenoid reductase (CruI) that produces chlorobactene derivatives [13,30,31] (Figure S1). Two types of 1,2-hydratase (CrtC and CruF) are known. CrtC was identified in purple bacteria [19]. CruF is present in the cyanobacterium *Picosynechococcus* sp. PCC 7002 (previously, *Synechococcus* sp. strain PCC 7002 [32]) and non-photosynthetic bacteria, *Deinococcus radiodurans* and *Deinococcus geothermalis* [33,34]. In aerobic bacteria, the synthesis of keto-carotenoids, which contain high reactive oxygen species scavenging activity [35,36], has also been observed. The ketolases responsible for keto-carotenoid synthesis include CrtO, CrtW, CrtA, and CruO [19,37,38,39,40,41].

**Figure 1:**
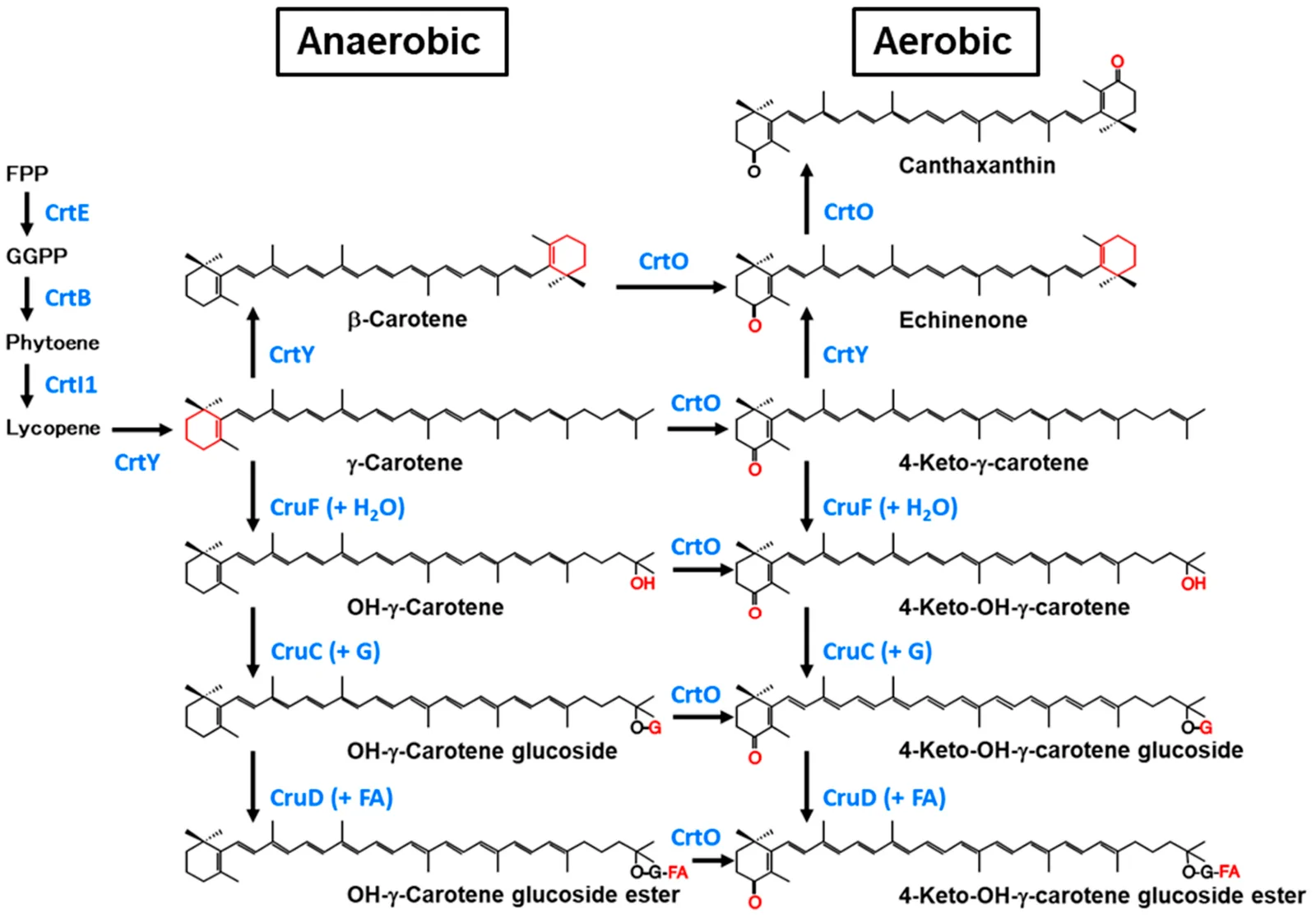
The proposed carotenoid biosynthetic pathway and synthetic enzymes found in *Cfx. aurantiacus*. γ-carotene, keto-γ-carotene, and their derivatives are produced in anaerobic and aerobic conditions. The pathway is proposed from the carotenoid structures [42], and the enzymes are identified in this study. Abbreviations: FPP, farnesyl pyrophosphate; FA, fatty acid; G, glucoside.

Filamentous anoxygenic phototrophic bacteria (FAPs) represent a diverse group of phototrophic microorganisms. *Chloroflexus* (*Cfx.*) *aurantiacus* contains chlorosomes, similar to GSB, however, it is a facultative anaerobe that uses a Type-II reaction center [43,44]. In contrast, *Roseiflexus* (*Rfx.*) *castenholzii* is a bacterium that lacks chlorosomes. Moreover, ‘*Candidatus* Chlorohelix allophototropha’ has recently been discovered and shown to possess chlorosomes, but utilizes a Type-I reaction center for photosynthesis [45]. Despite this diversity, studies of carotenoids in these organisms are limited. Although *Cfx. aurantiacus* mainly synthesizes OH-γ-carotene glucoside ester, as observed in *Cba. tepidum* [14,42] (Figure 1 and Figure S1), it is shown to produce keto-carotenoids [46]. Moreover, *Rfx. castenholzii* synthesizes carotenoids, such as methoxy-keto-myxocoxanthin and keto-myxocoxanthin glucoside ester [47]; however, the biosynthetic pathways for these compounds have not been elucidated. Chlorosomes of *Cfx. aurantiacus* contain approximately 25% (mol/mol) of carotenes per BChl [9]. On the other hand, the exact localization of carotenoids in the reaction centers of these bacteria remains unknown, even though no carotenoids were observed in the cryo-EM structure of the reaction center [48,49,50,51].

To shed light on the presence and biosynthesis of carotenoids in FAPs, we examined carotenoid synthesis in *Cfx. aurantiacus* to determine which molecular species of carotenoids are synthesized under different culture conditions. We searched the genome for candidate carotenoid biosynthesis genes based on the predicted final products and performed functional analyses by expressing these candidate genes in *E. coli*. Finally, by examining the transcriptional regulation of these genes under different culture conditions, we determined the selective production of different carotenoid molecular species in this bacterium.

## 2. Materials and Methods

### 2.1. Bacterial Strains and Culture Conditions

*Chloroflexus aurantiacus* strain J-10-fl [52] was grown anaerobically or aerobically under illumination or aerobically in the dark at 45 °C in YE medium [53]. *E. coli* strains JM109 and JM101 were used for cloning and analyzing carotenoid genes, respectively, and they were cultivated aerobically at 37 °C in LB medium or SOB medium [54].

### 2.2. Plasmid Constructs

The plasmids used in this study are listed in Table S1. The PCR primers used for plasmid construction are listed in Table S2. To overexpress carotenogenesis genes from *Chloroflexus aurantiacus* in *E. coli* JM101, eleven plasmids were constructed. To create pT-crtEB, pT-groE [55] was digested with NdeI and BamHI to remove a 2.04-kbp fragment containing *groES* and *groEL*. The *crtE* and *crtB* genes were amplified by PCR using the Cfx_E F/Cfx_E R and Cfx_B F/Cfx_B R primer sets, respectively. The two PCR products were assembled with the NdeI- and BamHI-digested pT-groE using the In-Fusion^®^ HD cloning kit (Takara, Shiga, Japane), to yield pT-crtEB. To construct the *crtI1*, *crtI2*, *crtI3*, *crtY*, and *crtO* expression vectors, the corresponding genes were amplified by PCR using the Cfx_I1 F/Cfx_I1 R, Cfx_I2 F/Cfx_I2 R, Cfx_I3 F/Cfx_I3 R, Cfx_Y F/Cfx_Y R, and Cfx_O F/Cfx_O R primer sets, respectively. The PCR products were cloned into the NdeI- and BamHI-digested pET21a(+) vector (Novagen/Merck KGaA, Darmstadt, Germany) using the In-Fusion cloning kit to generate ET21-crtI1, pET21-crtI2, pET21-crtI3, pET21-crtY, and pET21-crtO. To construct pT-crtEBI1, the *crtE*, *crtB*, and *crtI1* genes were amplified using the primer sets Cfx_E F/Cfx_E R, Cfx_B F/Cfx_B R2, and I1 for EB F/I1 for EB R, respectively. The three PCR products were assembled into the NdeI- and BamHI-digested pT-groE using the In-Fusion kit, generating pT-crtEBI1. pT-crtEBI1 was then used as a template for PCR with the pTcrtEBI1 F/pTcrtEBI1 R primer set. This fragment was assembled after amplifying the *crtY* fragment using Y for EBI1 F/Y for EBI1 R to yield pT-crtEBI1Y. To construct pRSF-cruF, the RSFDuet-1 vector (Novagen) was digested with NcoI and BamHI. The *cruF* gene was amplified using Cfx_cruF F/Cfx_cruF R, and the PCR product was assembled into the digested vector using the In-Fusion cloning kit to yield pRSF-cruF. To construct pRSF-cruFC, the *cruF* and *cruC* genes were amplified using Cfx_cruF F/Cfx_cruF R2 and Cfx_cruC F/Cfx_cruC R, respectively, and assembled into NcoI- and BamHI-digested RSFDuet-1. To construct pRSF-cruFCD, the *cruF*, *cruC*, and *cruD* genes were amplified using the Cfx_cruF F/Cfx_cruF R2, Cfx_cruC F/Cfx_cruC R2, and Cfx_cruD F/Cfx_cruD R primer sets, respectively, and sequentially assembled into the NcoI and BamHI sites of RSFDuet-1 in this order, yielding pRSF-cruFCD.

### 2.3. Determination of Pigment Compositions of Chloroflexus aurantiacus and E. coli

Pigments were extracted from the wet cells of *Chloroflexus aurantiacus* and *E. coli* using a mixture of acetone and methanol (7:2, *v*/*v*). Total carotenoids were analyzed using a μBondapak C_18_ column (RCM-type, Waters Milford, MA, USA) eluted with methanol (1.8 mL/min). For carotenes from *E. coli*, a NOVA-Pak C_18_ column (RCM-type, Waters) was used, and they were eluted with acetonitrile/tetrahydrofuran/methanol (58:7:35, by vol., 1.8 mL/min). Carotenoids were identified by HPLC retention times and absorption spectra compared with those in the wild type [42].

### 2.4. Phylogenetic Analysis of Phytoene Desaturase

A phylogenetic tree was constructed with translated sequences of CrtI, CrtP, and CrtQ paralogs using the MAFFT [56] and IQ-TREE [57] programs. The accession numbers for the sequences used to construct the tree were as follows: ‘*Candidatus* Chlorohelix allophototropha’ CrtI1 (WJW69520), CrtI2 (WJW66599), CrtI3 (WJW69748), CrtI4 (WJW66169); *Cereibacter sphaeroides* CrtI (Accession No. AXC61658); *Chloracidobacterium thermophilum* CrtH (AEP10865), CrtP (AEP12858), and CrtQ (AEP10819); *Chlorobaculum tepidum* CrtH (AAM71888), CrtP (AAM72043), and CrtQ (AAM72642); *Chloroflexus aurantiacus* CrtI1 (ABY35631), CrtI2 (ABY34641), and CrtI3 (ABY35619); *Gemmatimonas phototrophica* CrtI (AMW05347); *Heliobacterium mobile* CrtN (AAC84034); *Pantoea ananatis* CrtI (WP_176017230); *Prosthecochloris aestuarii* CrtH (ACF45807), CrtP (ACF45928), and CrtQ (ACF46544); *Rhodobacter capsulatus* CrtI (ADE84444); *Rhodopseudomonas palustris* CrtI (WCL91652); *Roseiflexus castenholzii* CrtI1 (ABU57678), CrtI2 (ABU59538), and CrtI3 (ABU58614).

### 2.5. Quantitative RT-PCR Assays for mRNA Quantitation

Expression of the carotenogenesis genes of *Chloroflexus aurantiacus* was determined using quantitative reverse transcription (RT)-PCR analysis of the mRNA. Gene fragments were amplified using the following primer sets (Table S3): *crtE*, bchE RT-F/-R; *crtB*, crtB RT-F/-R; *crtI1*, crtI1 RT-F/-R; *crtI2*, crtI2 RT-F/-R; *crtI3*, crtI3 RT-F/-R; *crtY*, crtY RT-F/-R; *cruF*, cruF RT-F/-R; *cruC*, cruC RT-F/-R; *cruD*, cruD RT-F/-R; *crtO*, crtO RT-F/-R; and *idi*, idi RT-F/-R. A fragment of the *rpoD* gene, which encodes the presumed major housekeeping sigma factor [58], was amplified using the rpoD RT-F and -R primers and was used as an internal standard. Total RNA was isolated from *Cfx. aurantiacus* cells grown to mid-log phase under aerobic or anaerobic illumination at 30 μE s^−1^ m^−2^, or aerobically in the dark, using a NuclepSpin^®^ RNAKit (MACHEREY-NAGEL (Düren, Germany)). The extracted mRNA was treated with RNase-free Recombinant DNaseI (Takara) to minimize genomic DNA contamination. Finally, cDNA was synthesized from the isolated RNA (30 ng per 10 μL of reaction mixture for reverse transcription reaction) using a TaKaRa RNA PCR Kit (AMV) Ver. 3.0 (Takara). PCR cycle conditions were as follows: 25 cycles for 30 s at 94 °C, 30 s at 63 °C, and 60 s at 72 °C. The PCR products were analyzed by 1.5% agarose gel electrophoresis.

### 2.6. Absorption Measurements of Whole Cells

UV/Vis/NIR absorption spectra were measured using a Hitachi U-3500 (High-technologies Corp., Tokyo, Japan). *Chloroflexus aurantiacus* cells were grown to stationary phase at 45 °C under aerobic or anaerobic incandescent illumination at 30 μE s^−1^ m^−2^, collected, suspended in 50 mM Tris-HCl (pH 7.8) buffer containing 150 mM NaCl, and measured.

## 3. Results

### 3.1. Carotenoid Compositions in Cfx. aurantiacus Cells Grown Under Various Conditions

Previous studies have examined the carotenoid composition of *Cfx. aurantiacus*. Takaichi et al. found that the pigments of strains J-10-fl and OK-70-fl under phototrophic conditions included γ-carotene, β-carotene, OH-γ-carotene, OH-γ-carotene glucoside, and OH-γ-carotene glucoside ester, as well as bacteriochlorophylls *a* and *c* [42]. Halfen et al. found that the strain J-10-fl also synthesized keto-carotenoids, such as echinenone and keto-γ-carotene, and bacteriochlorophylls *a* and *c*, without esterified carotenoids due to saponification [46]. Thus, *Cfx. aurantiacus* synthesizes a wide variety of carotenoids; it was considered that these carotenoid compositions might vary depending on the culture conditions. Indeed, the whole-cell absorption spectra indicated that cells grown under anaerobic light conditions formed chlorosomes, whereas this was barely observed under aerobic conditions, suggesting a major reorganization of the photosynthetic apparatus (Figure 2). To address how this cellular shift affects the diverse carotenoid composition of *Cfx. aurantiacus*, we analyzed its carotenoid profile under these distinct conditions (Figure 3).

When *Cfx. aurantiacus* was cultured under anaerobic light conditions, OH-γ-carotene, γ-carotene, and β-carotene were the major components. The synthesis of OH-γ-carotene and OH-γ-carotene glucoside ester derivatives was also observed (Figure 3a). Under this condition, no keto-carotenoids were observed. In contrast, when this bacterium was cultured under aerobic dark conditions, the carotenoid composition changed significantly, with the keto-carotenoids canthaxanthin and OH-γ-carotene as the major components: OH-γ-carotene glucoside, keto-OH-γ-carotene, keto-OH-γ-carotene glucoside ester, β-carotene, and OH-γ-carotene glucoside ester (Figure 3b). Under aerobic light conditions, the carotenoid composition of the cells was mostly unchanged compared with that under aerobic-dark conditions (Figure 3c). Therefore, *Cfx. aurantiacus* alters its carotenoid composition depending on culture conditions, particularly in the presence of oxygen.

### 3.2. Carotenoid Genes in the Cfx. aurantiacus Genome

Since the complete genome sequence has been determined [59] (Accession No. CP000909), we examined the genes encoding the enzymes responsible for carotenoid biosynthesis. We conducted bacterium-specific BLAST analysis, a program provided by the National Center for Biotechnology Information (https://www.ncbi.nlm.nih.gov/datasets/taxonomy/324602/, accessed on 3 August 2026), using functional genes (Table 1). We found that Caur_3742, Caur_2684, Caur_1819, Caur_0214, Caur_3844, Caur_0213, and Caur_1337 were candidates for *crtE*, *crtB*, *crtY*, *cruF*, *cruC*, *cruD*, and *crtO*, respectively. Interestingly, three paralogous genes of phytoene desaturase CrtI, Caur_2422, Caur_1413, and Caur_2410, were present in the genome of this bacterium. These candidate biosynthesis genes are scattered throughout the *Cfx. aurantiacus* genome, except for *cruD* and *cruF*, which are arranged in this order. We examined their function by heterologously expressing them in *E. coli* and analyzing the resulting pigments.

### 3.3. Functional Analyses of Three crtI Genes

We examined three *crtI* paralogs present in *Cfx. aurantiacus*, Caur_2422, Caur_1413, and Caur_2410, as *crtI1*, *crtI2*, and *crtI3*, respectively. Phylogenetic analysis of the predicted amino acid sequences revealed that they belong to distinct clades (Figure 4). *crtI1* belongs to the same clade as *crtI* from the purple bacteria *Cereibacter* (*Ceb.*) *sphaeroides* (previously, *Rhodobacter sphaeroides* [62]), *Rhodobacter capsulatus*, *Rhodopseudomonas palustris*, and a non-photosynthetic bacterium *Pantoea* (*P.*) *ananatis*, which function as phytoene desaturases [22,23,24,63,64]. On the other hand, *crtI2* belongs to the same clade as *crtN* [65], a diapo-phytoene desaturase involved in C30-carotenoid biosynthesis in *Heliobacterium mobilis*, whereas *crtI3* belongs to a clade of *crtI* paralogs from other bacteria with unknown functions. To determine the function of these CrtI enzymes, we expressed them in *E. coli*.

Many studies have been conducted on the heterologous expression of carotenoid synthesis genes in *E. coli* to examine their functions [22,24,39,66]. To determine whether the carotenoid synthase genes from *Cfx. aurantiacus* function in *E. coli*, we first introduced the *crtE* and *crtB* genes to verify whether phytoene, the starting material for carotenoid synthesis, could be synthesized (Figure 5a). The results confirmed phytoene synthesis, verifying that CrtE and CrtB encode the GGPP synthase and phytoene synthase, respectively.

Next, we determined whether each of the three *crtI* genes (Table 1) exhibited phytoene desaturase activity in the presence of CrtE and CrtB (Figure 5b–d). Lycopene synthesis was confirmed only in *E. coli* expressing *crtI1*, confirming that this enzyme is a phytoene desaturase. Although *crtI2* and *crtI3* did not exhibit this activity, it is unclear what role these enzymes play in *Cfx. aurantiacus*.

### 3.4. Function of the crtY Gene

Because *Cfx. aurantiacus* synthesizes cyclic carotenoids, such as β-carotene, γ-carotene, and canthaxanthin (Figure 3), it was expected to contain lycopene cyclase. Three types of lycopene cyclase are known, including CrtY/CrtL, CrtYc/CrtYd, and CruA/CruP; however, only a *crtY* paralog was present in the *Cfx. aurantiacus* genome (Table 1). Therefore, we expressed this gene under a lycopene synthesis background, introducing the *crtE*, *crtB*, and *crtI1* genes of *Cfx. aurantiacus*, which were examined above, into *E. coli* to identify the carotenoids synthesized (Figure 6b). The results confirmed the synthesis of β-carotene and γ-carotene. Thus, CrtY from *Cfx. aurantiacus* is capable of synthesizing β-carotene from lycopene via γ-carotene on its own.

### 3.5. Expression of cruF, cruC, and cruD Genes in E. coli

As *Cfx. aurantiacus* synthesizes OH-γ-carotene glucoside ester, this bacterium expresses enzymes capable of synthesizing the OH-γ-carotene glucoside ester from γ-carotene. Therefore, the paralogs of *cruF*, *cruC*, and *cruD* are present in its genome (Table 1). To determine the function of *cruF* as a carotenoid 1,2-hydratase, we firstly co-expressed *cruF* with *crtE*, *crtB*, *crtI1*, and *crtY* genes in *E. coli* (Figure 7b). OH-γ-carotene was detected, indicating the function of *cruF* as a hydratase. In addition, di-OH-lycopene, as well as β-carotene, was synthesized, and we found that CruF was also able to react with lycopene. We hypothesized that in recombinant *E. coli* cells, the CruF and CrtY enzymes were competing for lycopene as a substrate. The results suggest that CrtY does not proceed with the cyclization of both sides of lycopene at once, but rather performs the reaction on one side at a time, followed by the release of the synthesized γ-carotene.

To examine the glucoside transferase activity of CruC, the *cruC* and *cruF* genes were heterologously co-expressed in *E. coli* under the same β-carotene synthesis conditions described above. OH-γ-carotene glucoside and di-OH-lycopene di-glucoside were detected (Figure 7c). This confirmed that CruC from *Cfx. aurantiacus* is a glucosyltransferase. Furthermore, CruC adds glucoside groups to both ends of di-OH-lycopene, which serves as a substrate.

Next, to study the fatty acid esterification activity of CruD, we co-expressed *cruF*, *cruC*, and *cruD* in *E. coli* with a background of β-carotene (Figure 7d). The production of OH-γ-carotene glucoside ester was confirmed, thus indicating the ability of CruD to add fatty acids. Di-OH-lycopene di-glucoside ester derivatives were also detected. These results indicate that OH-γ-carotene glucoside ester is synthesized within *Cfx. aurantiacus* cells using γ-carotene as a substrate through the activity of CruF, CruC, and CruD.

### 3.6. Function of CrtO as Ketolase in Cfx. aurantiacus

The synthesis of keto-carotenoids was observed when *Cfx. aurantiacus* was cultured under aerobic conditions, suggesting that this bacterium expresses a functional ketolase (Figure 3). Genome analysis revealed a paralog of *crtO*, which is a known β-carotene ketolase. To characterize its function, this gene was expressed in *E. coli* with a β-carotene synthesis background (Figure 8b). Consequently, the synthesis of echinenone was confirmed, indicating that CrtO possesses ketolase activity. Although canthaxanthin was the predominant carotenoid in the aerobic culture of *Cfx. aurantiacus*, it was not detected in the recombinant *E. coli*. The yield of echinenone was also low, which may be attributed to the low activity of CrtO during heterologous expression, thus preventing canthaxanthin from accumulating at detectable levels. Furthermore, the *crtO* gene was introduced into *E. coli* strains with backgrounds for synthesizing OH-γ-carotene, OH-γ-carotene glucoside, and OH-γ-carotene glucoside ester, respectively. Although trace amounts of keto-carotenoids were detected by HPLC, individual identification was difficult. This limitation may result from the low activity of CrtO in the *E. coli* heterologous system, which is consistent with the results in Figure 8b. Because the wild-type strain accumulated keto-carotenoids (Figure 3), CrtO is expected to be fully active in its native environment (Figure 1). The *crtO* deletion mutants of cyanobacteria *Synechocystis* sp. PCC 6803 and *Anabaena* sp. PCC 7120 did not produce keto-carotenoids during photosynthetic growth [37,40,67]. Since both BChl *a* and BChl *c* were absent in Figure 3b,c, the reactive oxygen species generated by BChls could not yield keto-carotenoids.

Moreover, we performed a semi-quantitative RT-PCR analysis of carotenogenesis genes using *Cfx. aurantiacus* cells grown under anaerobic-light, aerobic-dark, or aerobic-light conditions (Figure 9). The transcription levels of *crtE*, *crtB*, *crtI1*, *crtI2*, *crtI3*, *crtY*, *cruF*, *cruC*, *cruD*, and *idi* were almost identical across the three culture conditions. In contrast, the expression of *crtO* was lower under anaerobic-light conditions than under aerobic-dark or aerobic-light conditions, suggesting that the transcription of *crtO* is regulated by the presence of oxygen.

## 4. Discussion

In this study, we identified carotenoid biosynthesis genes in the genome of *Cfx. aurantiacus* and characterized their functions by heterologous expression in *E. coli*. The results indicated that CrtE, CrtB, CrtI1, CrtY, CruF, CruC, CruD, and CrtO are functional components of the carotenoid biosynthetic pathway. The proposed pathway involving these enzymes in *Cfx. aurantiacus* is illustrated in Figure 1. Specifically, *Cfx. aurantiacus* produces OH-γ-carotene glucoside ester via CruF, CruC, and CruD, whereas *Cba. tepidum* synthesizes it through CrtC, CruC, and CruD. Thus, these bacteria use different types of carotene 1,2-hydratases, CruF and CrtC, while sharing the same glucosidase (CruC) and esterase (CruD) (see also Figure S1).

*Cfx. aurantiacus* is capable of growth under anaerobic photosynthetic and aerobic respiratory conditions. This metabolic versatility likely reflects the presence of adaptive regulatory systems corresponding to each growth mode. Under photosynthetic conditions, *Cfx. aurantiacus* forms chlorosomes (Figure 2). Although GSBs also form chlorosomes, they are strict anaerobes. When exposed to oxygen and high levels of reactive oxygen species, the chlorosomes of GSBs quench absorbed light energy mediated by quinones, which is thought to protect cells from oxidative stress [7,68,69,70]. In contrast, such light-energy quenching systems have not been observed in the chlorosomes of *Cfx. aurantiacus*. Kim et al. [17] examined the role of carotenoids in protection against photo-oxidative damage in *Cba. tepidum* using carotenoid mutants. They concluded that, in this strictly anaerobic bacterium, the protective effect of carotenoids within the chlorosome is limited for the self-assembly of BChl *c*, which form the light-harvesting core. Instead, carotenoid protection is more effective at quenching the triplet state of BChl *a* bound to the CsmA baseplate protein. Yakovlev and Taisova [71] found that in *Cfx. aurantiacus*, the formation rate of the carotenoid triplet state in CsmA is sufficient to fully quench the triplet state of BChl *a*, thereby inhibiting the generation of singlet oxygen. Consequently, in *Cfx. aurantiacus* cells, carotenoids may act as quenchers of the triplet BChl *a* state in the baseplate, while directly scavenging generated reactive oxygen species.

When *Cfx. aurantiacus* is cultured in the presence of oxygen, the synthesis of keto-carotenoids occurs (Figure 3). Because keto-carotenoids are not detected in anaerobic photosynthetic cultures, their synthesis is likely strictly regulated. Examination of the transcript levels of the carotenoid biosynthesis genes using RNA extracted from cells cultured under anaerobic-light and aerobic-dark conditions revealed that *crtO* transcription was significantly upregulated under aerobic conditions (Figure 9). Similar results were obtained in a proteomic analysis of *Cfx. aurantiacus*, where CrtO expression was downregulated alongside the cellular transition to phototrophic growth (anaerobic light conditions) [72]. This indicates that the enhanced expression of CrtO in the presence of oxygen promotes keto-carotenoid synthesis. Because keto-carotenoids exhibit strong antioxidant activity, they may protect *Cfx. aurantiacus* from photo-oxidative damage. Conversely, the absorption spectra indicate that cells grown under aerobic conditions do not form chlorosomes containing BChl c, which serves as a source of reactive oxygen species (Figure 2). Therefore, in addition to the protective role of keto-carotenoids, *Cfx. aurantiacus* may repress chlorosome assembly under aerobic conditions to avoid reactive oxygen species production altogether. These results highlight the coordinated metabolic adaptation of *Cfx. aurantiacus* to both aerobic and photosynthetic environments.

By characterizing *Cfx. aurantiacus* in the present study, we obtained insight into carotenoid biosynthesis in FAPs. We demonstrated that among the three *crtI* paralogs present in the genome of *Cfx. aurantiacus*, only *crtI1* functions as a phytoene desaturase. This supports the hypothesis that FAPs containing a Type-II reaction center harbor a CrtI enzyme, which is consistent with other photosynthetic bacteria. In the phylogenetic tree shown in Figure 4, one of the candidate *crtI* genes from *Roseiflexus castenholzii* clustered closely with that of *Cfx. aurantiacus*. This suggests that *Rfx. castenholzii* utilizes CrtI. Interestingly, ‘*Candidatus* Chlorohelix allophototropha’, which performs photosynthesis via a Type-I reaction center, also contains a CrtI that is phylogenetically close to that of *Cfx. aurantiacus*. In contrast, *Heliobacterium mobilis*, which also harbors a Type-I reaction center, contains CrtN, a C30 carotenoid desaturase. Although this enzyme shows sequence homology with CrtI, it forms a distinct clade in the phylogenetic tree. If the CrtI of ‘*Candidatus* Chlorohelix allophototropha’ is confirmed to function as a phytoene desaturase, it may provide deeper insight into carotenoid biosynthesis and the evolutionary history of the reaction centers. Therefore, future studies should focus on the functional characterization of this CrtI.

## 5. Conclusions

In this study, we examined the carotenoid biosynthetic system of *Cfx. aurantiacus* by combining pigment analysis, genome mining, heterologous expression in *E. coli*, and transcriptional profiling. Under anaerobic phototrophic and aerobic heterotrophic conditions, *Cfx. aurantiacus* exhibited a distinct carotenoid composition, indicating that carotenoid production is environmentally regulated, particularly by oxygen availability. Genome analysis revealed candidate genes involved in the synthesis of phytoene, lycopene, cyclic carotenoids, glucosylated carotenoids, and keto-carotenoids. Functional characterization revealed that CrtI1 is a phytoene desaturase, CrtY converts lycopene to γ-carotene and β-carotene, CruF acts as a carotenoid 1,2-hydratase, CruC functions as a carotenoid glucosyltransferase, CruD participates in esterification of glucosylated carotenoids, and CrtO has β-carotene ketolase activity. Based on these results, we propose a carotenoid biosynthetic pathway that leads to the major carotenoids detected in *Cfx. aurantiacus*. Our results also suggest that the oxygen-dependent regulation of *crtO* transcription contributes to the selective production of keto-carotenoids under aerobic conditions, which likely enhance protection against oxidative stress. These results provide insight into the diversity and regulation of carotenoid metabolism in FAPs. In particular, the identification of a functional CrtI in *Cfx. aurantiacus* supports the broader view that Type-II reaction center-containing photosynthetic bacteria use CrtI-mediated lycopene biosynthesis. Overall, the results of this study advance our understanding of carotenoid biosynthesis, physiological adaptation, and the evolutionary diversity of photosynthetic bacteria.

## Figures and Tables

**Figure 2:**
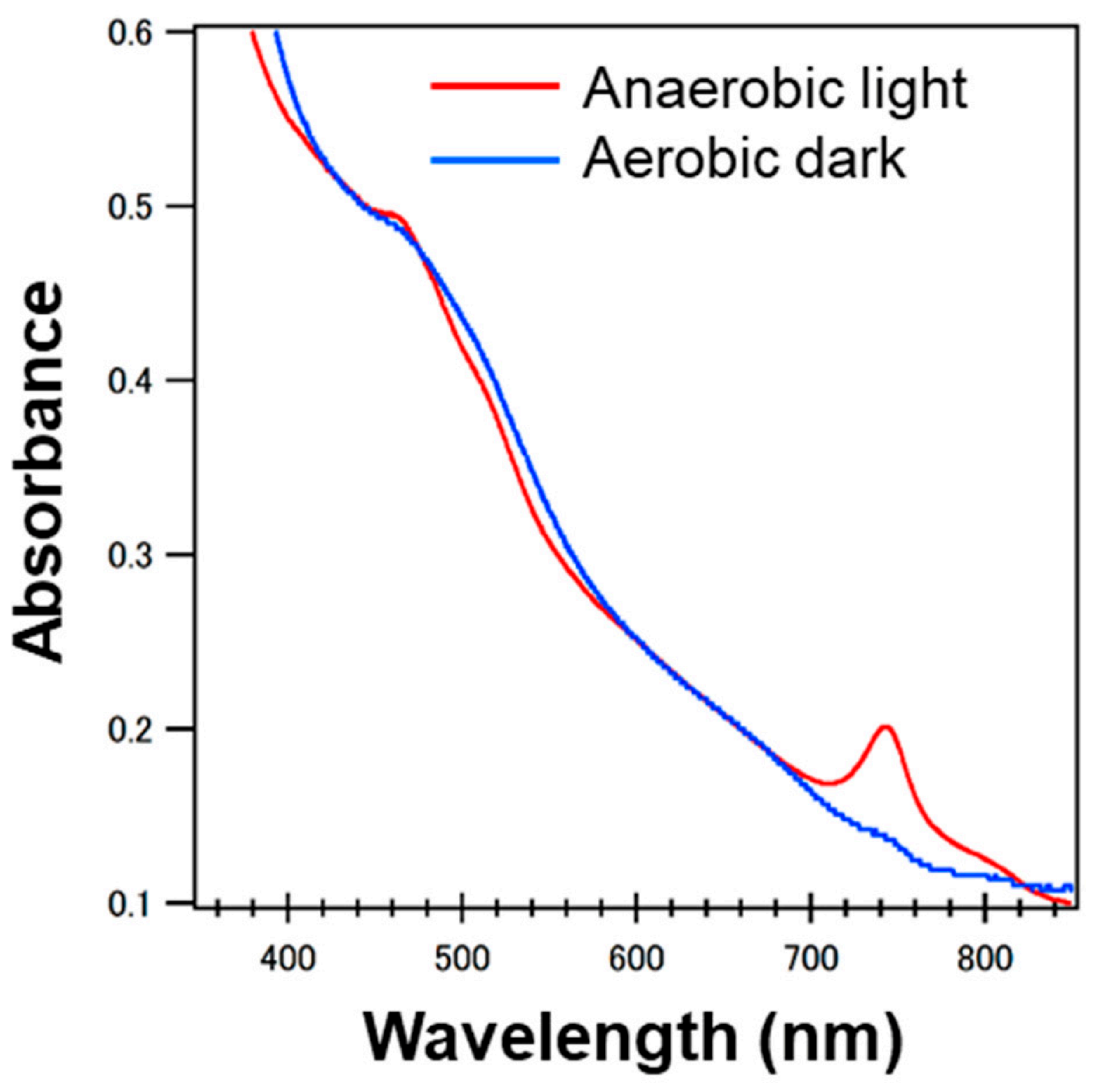
Absorption spectrum measurements of *Cfx. aurantiacus* cells under anaerobic light (red) and aerobic dark (blue) conditions.

**Figure 3:**
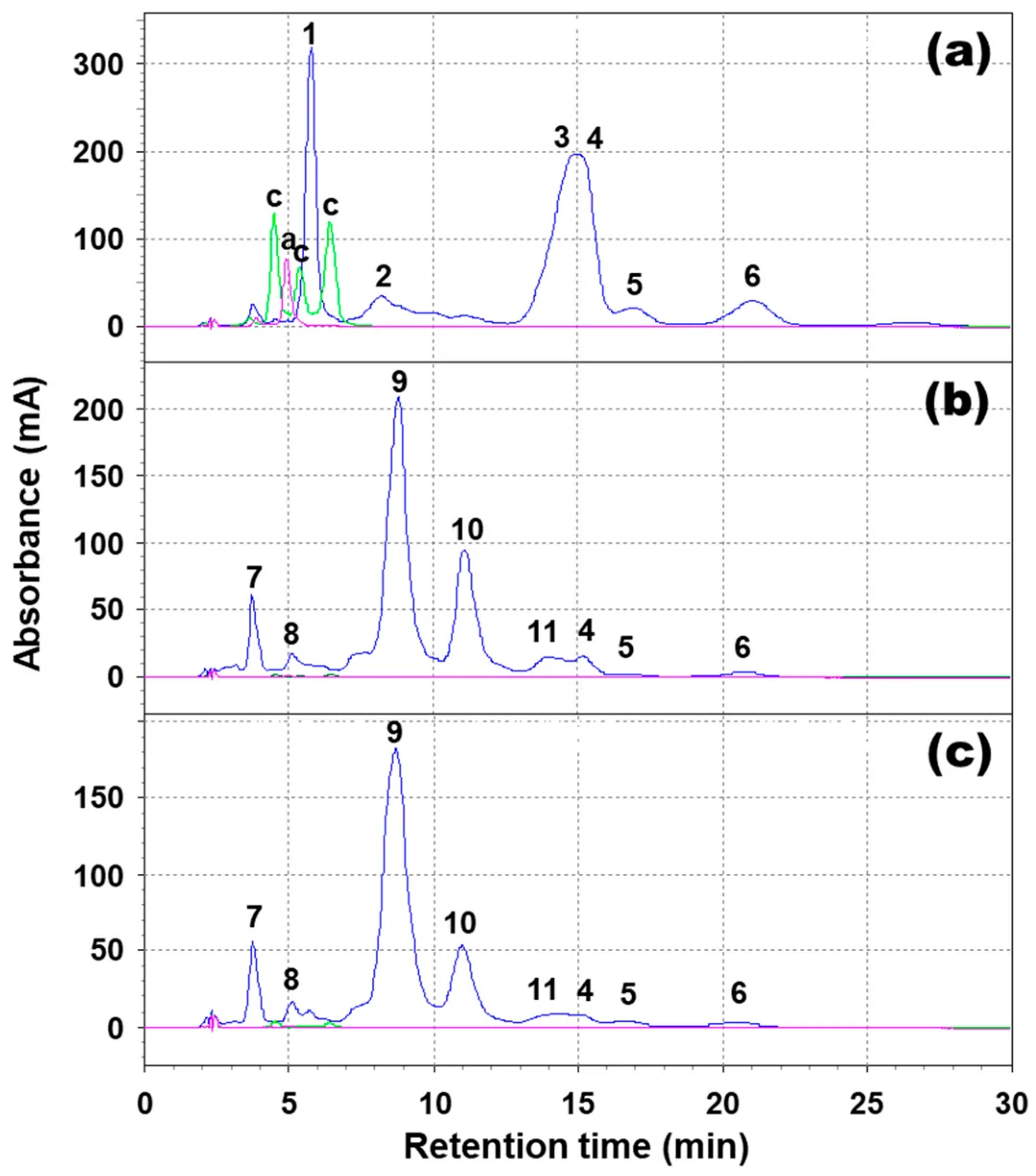
HPLC measurements of extracted pigments from *Cfx. aurantiacus* cells grown under anaerobic light (**a**), aerobic dark (**b**) and aerobic light (**c**) conditions. Cells were inoculated at a 1:1000 dilution from an anaerobic light-grown preculture and cultivated under each respective condition. A μBondapak C_18_ column was used, and the blue, green, and magenta lines show measurements at 470 nm for carotenoids, 669 nm for bacteriochlorophyll *c* homologs, and 770 nm for bacteriochlorophyll *a*, respectively. Absorption spectra of major carotenoids in each culture condition are shown in Figure S2. a, bacteriochlorophyll *a*; c, bacteriochlorophyll *c*. Peak 1, OH-γ-carotene glucoside; peak 2, OH-γ-carotene; peak 3, γ-carotene; peak 4, β-carotene; peak 5, OH-γ-carotene glucoside ester; peak 6, OH-γ-carotene glucoside ester; peak 7, keto-OH-γ-carotene glucoside; peak 8, keto-OH-γ-carotene; peak 9, canthaxanthin; peak 10, keto-γ-carotene; and peak 11, keto-OH-γ-carotene glucoside ester.

**Figure 4:**
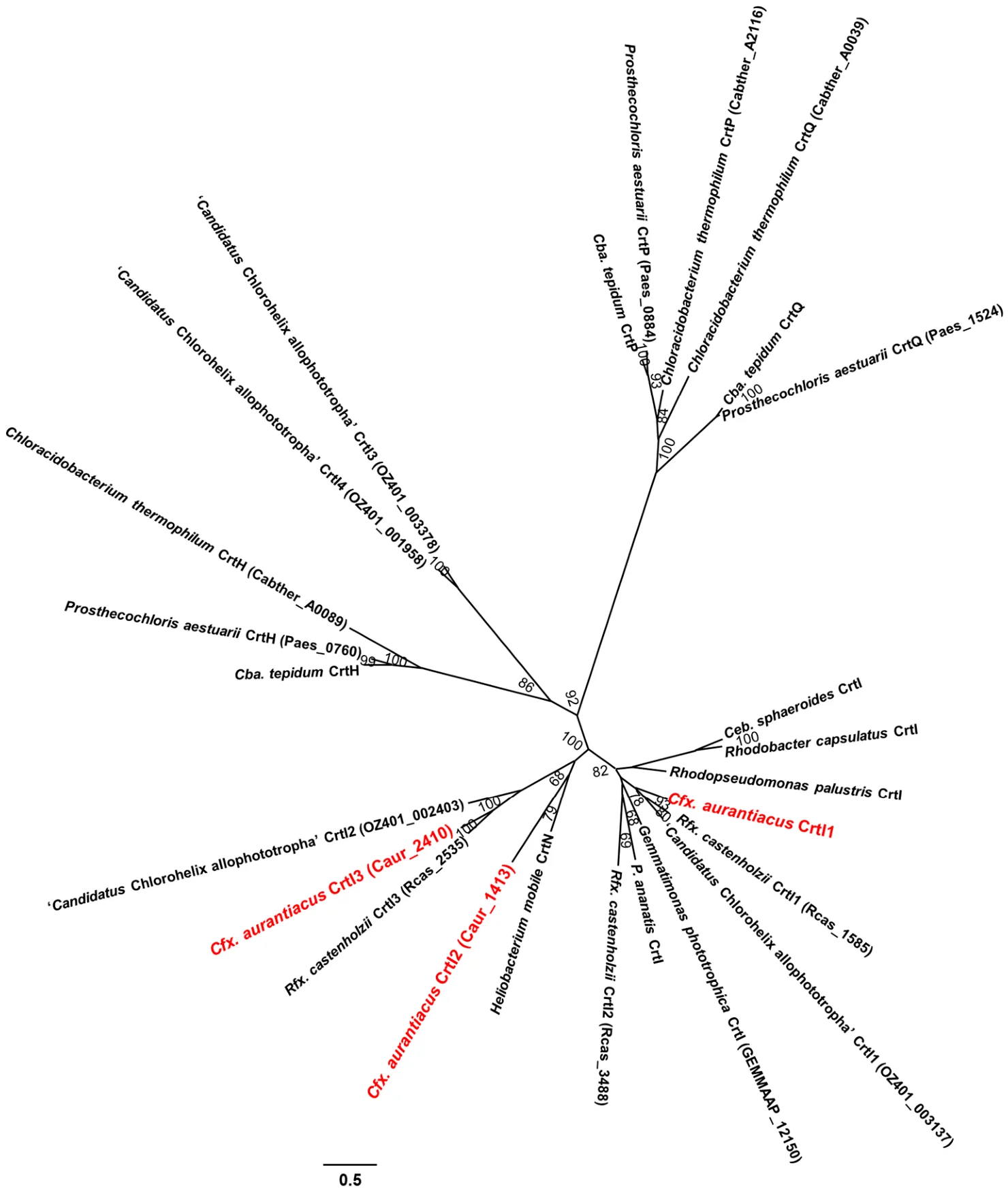
Phylogenetic analysis of *crtI* paralogs among photosynthetic bacteria. The *crtI* of *Pantoea ananatis* was used as a control for non-photosynthetic bacteria. For proteins that did not undergo functional analysis, a gene locus tag was assigned.

**Figure 5:**
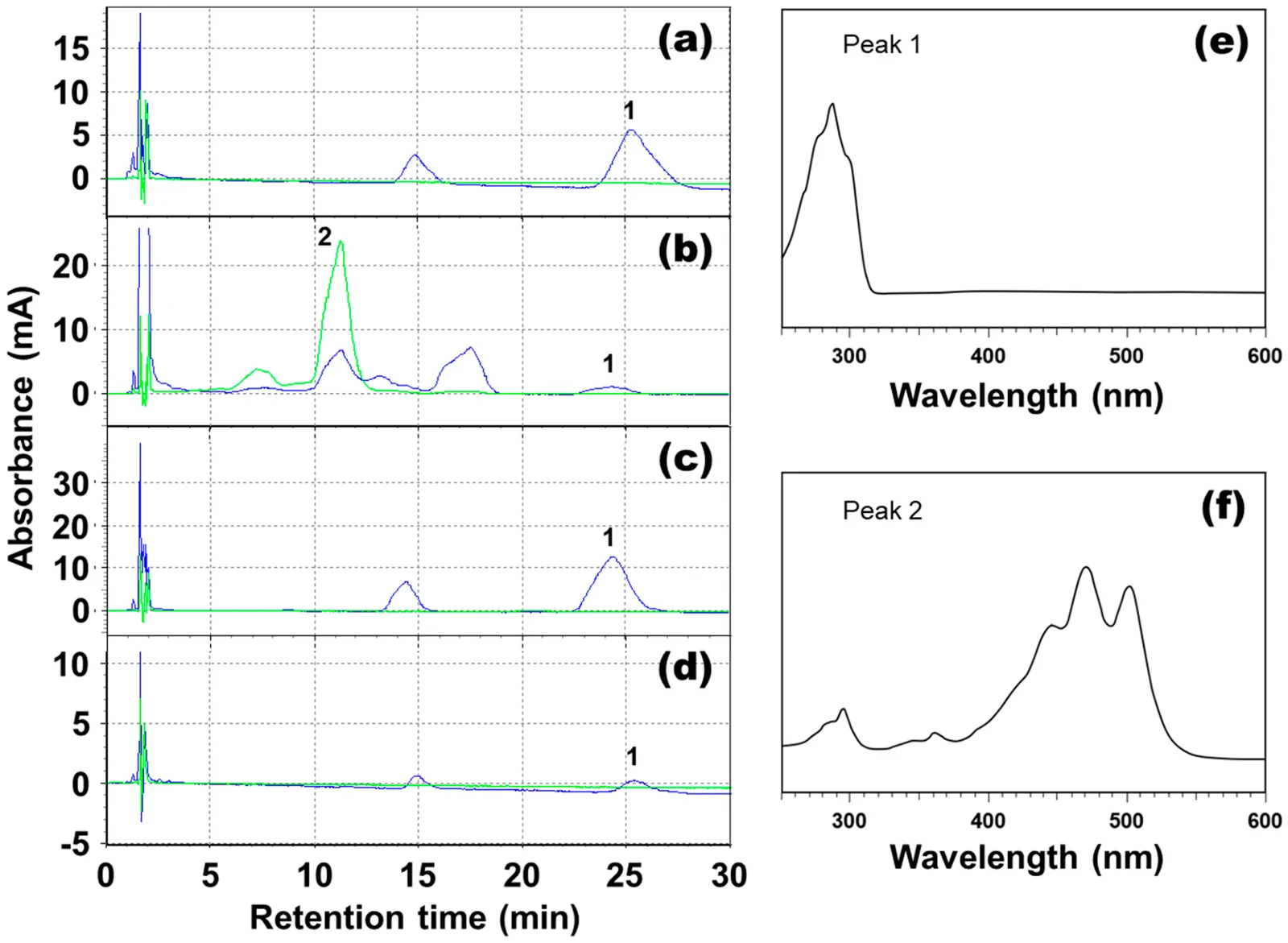
HPLC elution profiles of carotenoids produced by expressing *Cfx. aurantiacus crtB*, *crtE*, *crtI1*, *crtI2*, and *crtI3* in *E. coli* (**a**–**d**). A NOVAPAC C_18_ column was used. Blue and green lines represent the absorbance at 288 and 504 nm, respectively. (**a**) Pigments from *E. coli* containing plasmid pT-crtEB. Pigments from *E. coli* containing pT-crtEB with the plasmid pET21-crtI1 (**b**), pET21-crtI2 (**c**), or pET21-crtI3 (**d**). Panels (**e**,**f**) show absorption spectra of peak 1 in panel (**a**) and peak 2 in panel (**b**), respectively. Peak 1, phytoene; peak 2, lycopene. Some impurities were also present.

**Figure 6:**
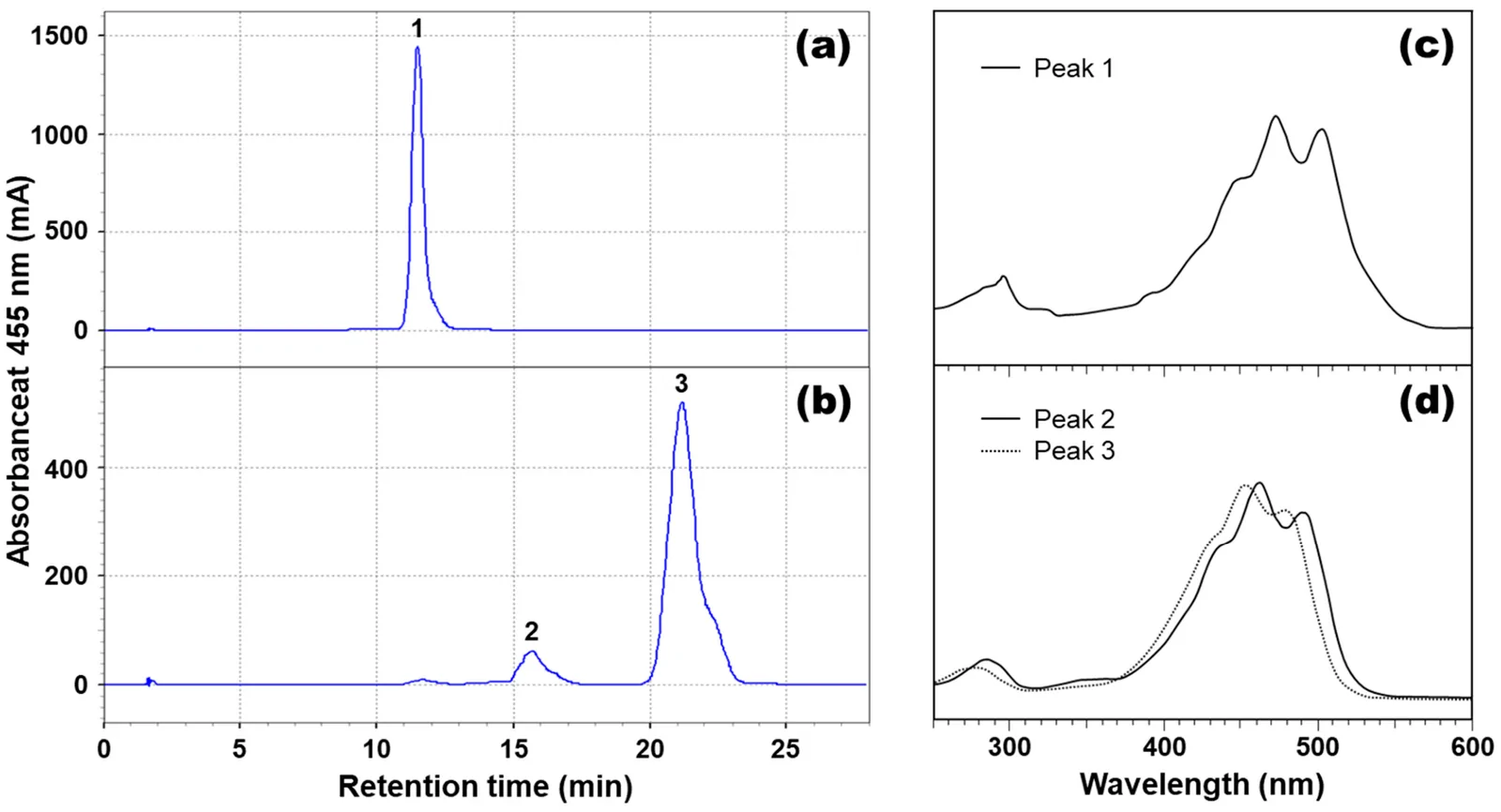
HPLC elution profiles of carotenoids produced following the expression of *Cfx. aurantiacus crtY* in *E. coli* with a lycopene background. A NOVAPAC C_18_ column was used, and absorbance at 485 nm is shown. (**a**) Pigments from *E. coli* containing only plasmid pT-crtEBI1. (**b**) Pigments from *E. coli* containing the plasmids pT-crtEBI1 and pET21-crtY. Panels (**c**,**d**) show absorption spectra of peak 1 in panel (**a**), and peaks 2 and 3 in panel (**b**), respectively. Peak 1, lycopene; peak 2, γ-carotene; peak 3, β-carotene.

**Figure 7:**
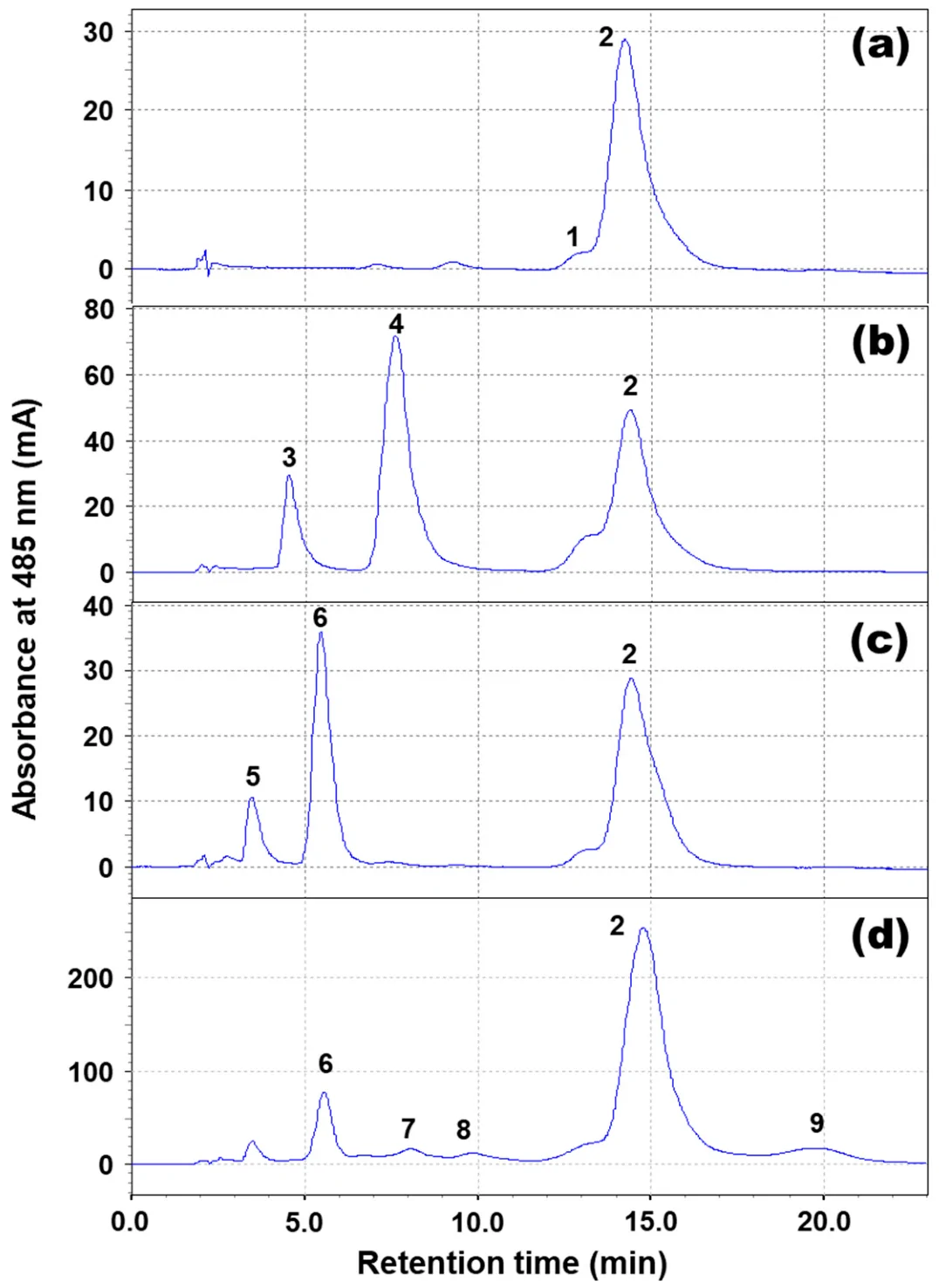
HPLC elution profiles of carotenoids produced from the expression of *Cfx. aurantiacus cruF*, *cruC*, and *cruD* in *E. coli* with a β-carotene background. A μBondapak C_18_ column was used, and the absorbance at 485 nm is shown. (**a**) Pigments from *E. coli* containing only plasmid pT-crtEBI1Y. Pigments from *E. coli* containing pT-crtEBI1Y with the plasmid pRSF-cruF (**b**), pRSF-cruFC (**c**), or pRSF-cruFCD (**d**). Absorption spectra of major carotenoids in each peak are shown in Figure S3. Peak 1, phytoene; peak 2, β-carotene; peak 3, di-OH-lycopene; peak 4, OH-lycopene; peak 5, di-OH-lycopene-di-glucoside; peak 6, OH-γ-carotene glucoside; peak 7, di-OH-lycopene di-glucoside ester; peak 8, di-OH-lycopene di-glucoside ester; and peak 9, OH-γ-carotene glucoside ester.

**Figure 8:**
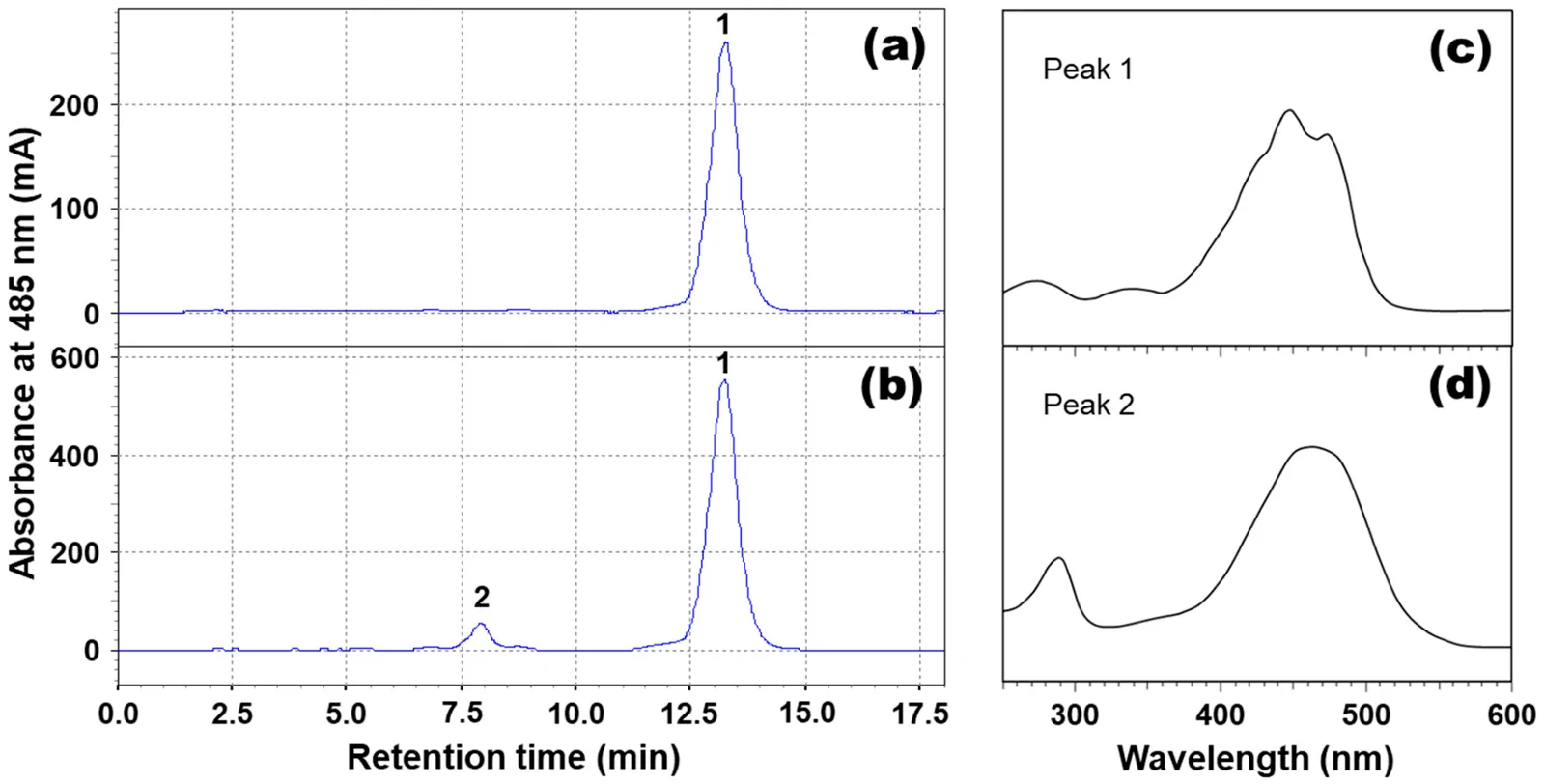
HPLC elution profiles of pigments produced by the expression of *Cfx. aurantiacus crtO* in *E. coli* with a β-carotene background. A μBondapak C_18_ column was used, and the absorbance at 485 nm is shown. (**a**) Pigments from *E. coli* containing only plasmid pT-crtEBI1Y. (**b**) Pigments from *E. coli* containing the plasmids pT-crtEBI1Y and pET-crtO. Panels (**c**,**d**) show absorption spectra of peak 1 in panel (**a**) and peak 2 in panel (**b**), respectively. Peak 1, β-carotene; peak 2, echinenone.

**Figure 9:**
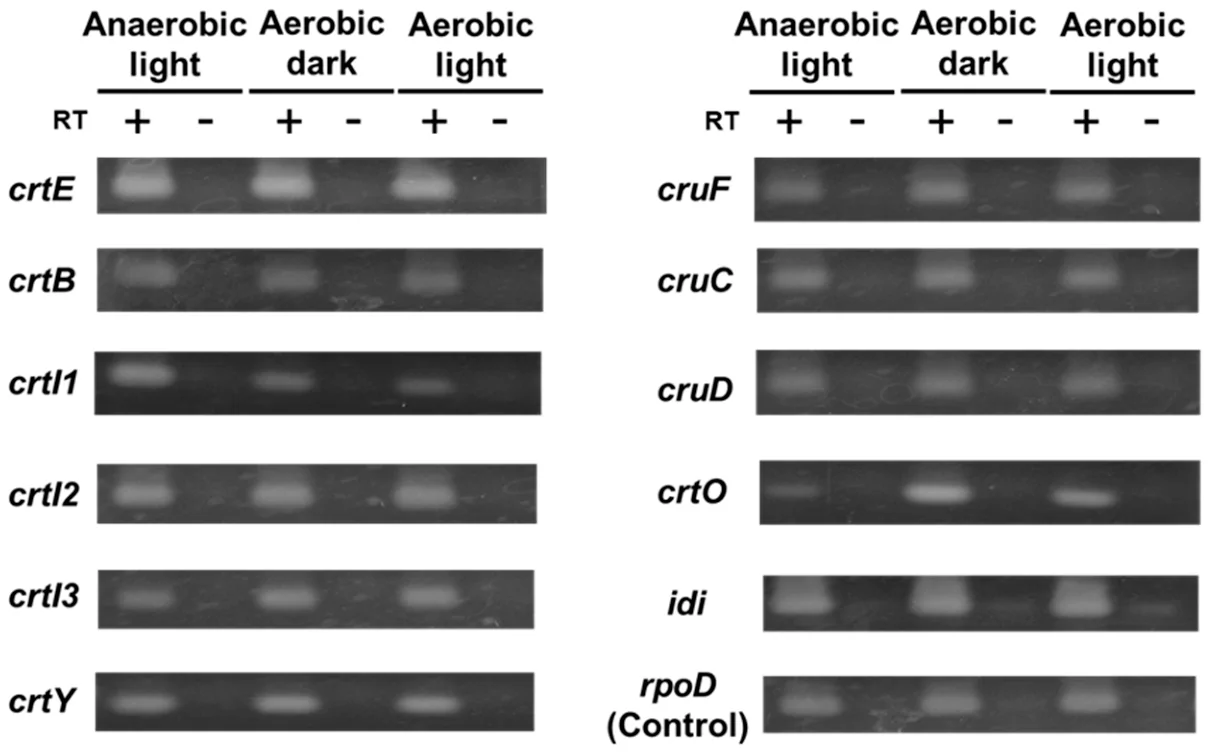
Semi-quantitative RT-PCR analysis of carotenoid biosynthetic gene transcripts with total RNA isolated from *Cfx. aurantiacus* wild-type cells grown under anaerobic light, aerobic dark, or aerobic light conditions. The *crtO* gene as well as the *crtE*, *crtB*, *crtI1*, *crtI2*, *crtI3*, *crtY*, *cruF*, *cruC*, *cruD*, and *idi* genes were analyzed. The *rpoD* gene encoding the major housekeeping sigma factor was used as an internal standard. Negative-control reactions omitted reverse transcriptase (RT −).

**Table 1:** Candidate carotenogenesis genes in *Cfx. aurantiacus*.

Genes	Enzymes	Query Sequences for BLAST ^1^		*Cfx. aurantiacus*	
		Accession No.	Organisms	Accession No. (Locus Tag)	Identity/e-Value
*crtE*	GGPP-synthase	AAR98495	*Bradyrhizobium* sp. ORS 278 [60]	ABY36920 (Caur_3742)	32%/1e-07
*crtB*	phytoene synthase	BAA14128	*Pantoea ananatis*^2^ [22]	ABY35889 (Caur_2684)	32%/3e-29
*crtI1*	phytoene desaturase	AXC61658	*Cereibacter sphaeroides*^3^ [23]	ABY35631 (Caur_2422)	40%/4e-100
*crtI2*		AXC61658	*Cereibacter sphaeroides*	ABY34641 (Caur_1413)	31%/1e-53
*crtI* *3*		AXC61658	*Cereibacter sphaeroides*	ABY35619 (Caur_2410)	29%/2e-41
*crtY*	lycopene cyclase	AAG28705	*Streptomyces griseus* [61]	ABY35036 (Caur_1819)	40%/5e-85
*cruF*	carotene 1,2-hydratase	ACB00019	*Picosynechococcus* sp. PCC 7002 ^4^ [33]	ABY33467 (Caur_0214)	31%/2e-09
*cruC*	glucosyltransferase	WP_010933643	*Chlorobaculum tepidum* [30]	ABY37021 (Caur_3844)	35%/1e-48
*cruD*	glucosyl esterase	WP_010932647	*Chlorobaculum tepidum* [30]	ABY33466 (Caur_0213)	49%/4e-25
*crtO*	β-carotene ketolase	BDH99146	*Synechocystis* sp. PCC 6803 [37]	ABY34565 (Caur_1337)	38%/6e-111

^1^ Query sequences were selected based on functional enzymes. ^2^ Formerly known as *Erwinia uredovora*. ^3^ Formerly known as *Rhodobacter sphaeroides*. ^4^ Formerly known as *Synechocuccus* sp. PCC 7002.

## Data Availability

The datasets supporting the conclusions of this study are available from the corresponding author upon reasonable request.

## Supplementary Materials

The following supporting information can be downloaded at https://www.mdpi.com/article/10.3390/genes17101189/s1, Figure S1: Carotenoid biosynthetic pathway of *Cba. tepidum*; Figure S2, Absorption spectra of major carotenoids from *Cfx. aurantiacus* detected in Figure 3; Figure S3, Absorption spectra of major carotenoids detected in Figure 7; Table S1: Plasmids used in this study; Table S2: Sequences of primers for construction of the plasmids used in this study; Table S3: Sequences of primers for the qRT-PCR assays used in this study.

## Abbreviations

The following abbreviations are used in this manuscript:

BChl	bacteriochlorophyll
*Ceb.*	*Cereibacter*
*Cba.*	*Chlorobium*
*Cfx.*	*Chloroflexus*
FAPs	filamentous anoxygenic photosynthetic bacteria
GGPP	geranylgeranyl pyrophosphate
GSBs	green sulfur bacteria
*P.*	*Pantoea*
RT	revers transcription
*Rfx.*	*Roseiflexus*
