# Supplementary material for: Functional Analysis of Carotenoid Biosynthetic Genes and Pathway of the Filamentous Anoxygenic Phototrophic Bacterium Chloroflexus aurantiacus
Source: Genes (Basel). 2026 Sep 25;17(10):1189. doi: 10.3390/genes17101189 (PMC13704108; doi:10.3390/genes17101189)
Supplement: Supplementary file 1 [file genes-17-01189-s001.zip › genes-4509554-supplementary.pdf]

Table S1. Plasmids used in this study.

Plasmids	Relevant characteristics	Reference or source
pT-groE	pACTC184 derivative used as an overexpression vector (Cm^r)	[1]
pET-21a(+)	an overexpression vector (Ap^r)	Novagen
RSFDuet-1	an overexpression vector (Km^r)	Novagen
pT-crtEB	DNA fragment containing *crtE* and *crtB* genes, cloned into the pT-groE in this order (Cm^r)	this work
pT-crtEBI1	DNA fragment containing *crtE*, *crtB*, and *crtI1* genes, cloned into the pT-groE in this order (Cm^r)	this work
pT-crtEBI1Y	DNA fragment containing *crtE*, *crtB*, *crtI1*, and *crtY* genes, cloned into the pT-groE in this order (Cm^r)	this study
pET21-crtI1	DNA fragment containing *crtI1* gene cloned into the pET21a(+) (Ap^r)	this study
pET21-crtI2	DNA fragment containing *crtI2* gene cloned into the pET21a(+) (Ap^r)	this study
pET21-crtI3	DNA fragment containing *crtI3* gene cloned into the pET21a(+) (Ap^r)	this study
pET21-crtY	DNA fragment containing *crtY* gene cloned into the pET21a(+) (Ap^r)	this study
pET21-crtO	DNA fragment containing *crtO* gene cloned into the pET21a(+) (Ap^r)	this study
pRSF-cruF	DNA fragment containing *cruF* gene cloned into the RSFDuet-1 (Km^r)	this study
pRSF-cruFC	DNA fragment containing *cruF* and *cruC* genes cloned into the RSFDuet-1 in this order (Km^r)	this study
pRSF-cruFCD	DNA fragment containing *cruF*, *cruC*, and *cruD* genes cloned into the RSFDuet-1 in this order (Km^r)	this study

The abbreviations: Ap^r, ampicillin resistance; Cm^r, chloramphenicol resistance; Km^r, kanamycin resistance.

Reference

1. Yasukawa, T.; Kanei-Ishii, C.; Maekawa, T.; Fujimoto, J.; Yamamoto, T.; Ishii, S. Increase of solubility of foreign proteins in *Escherichia coli* by coproduction of the bacterial thioredoxin. *J. Biol. Chem.* **1995**, *270*, 25328–25331.

Table S2. Sequences of primers for construction of the plasmids used in this study.

Primer name	Primer sequence	Overlapped sequence for In-Fusion reaction (uppercase letters)
Cfx_E F	GAAGGAGATATACATatggccggctaccagttac	overlapped with *Nde*I site of pT-groG
Cfx_E R	ctacaccgagcgctcaagc	
Cfx_B F	GAGCGCTCGGTGTAGcggcattatcaccatgtcct	overlapped with Cfx_E R primer
Cfx_B R	CGATAAGCTTGGATCttaccgcgataacttcttcgt	overlapped with *Bam*HI site of pT-groG
Cfx_I1 F	AGAAGGAGATATACATatggggcagaaaaggattgtc	overlapped with *Nde*I site of pET21a(+)
Cfx_I1 R	GCTCGAATTCGGATCCgagctcacgcctgttcctggctt	overlapped with *Bam*HI site of pET21a(+)
Cfx_I2 F	AGAAGGAGATATACATgtgtcaccacgaattgttattg	overlapped with *Nde*I site of pET21a(+)
Cfx_I2 R	GCTCGAATTCGGATCCgagctcaatgggcaaccgcggttgt	overlapped with *Bam*HI site of pET21a(+)
Cfx_I3 F	AGAAGGAGATATACATttgacgatgccgtgggtga	overlapped with *Nde*I site of pET21a(+)
Cfx_I3 R	GCTCGAATTCGGATCCgagctcagacaggcatatcagccgc	overlapped with *Bam*HI site of pET21a(+)
Cfx_Y F	GAAGGAGATATACATatggactacgactatatcattg	overlapped with *Nde*I site of pET21a(+)
Cfx_Y R	GCTCGAATTCGGATCtcaccccatctcccactcg	overlapped with *Bam*HI site of pET21a(+)
Cfx_O F	GAAGGAGATATACATatggcagcaacgagcgact	overlapped with *Nde*I site of pET21a(+)
Cfx_O R	GCTCGAATTCGGATCCttagtcccgcagtagtttgc	overlapped with *Bam*HI site of pET21a(+)
Cfx_B R2	ttaccgcgataacttcttcgt	
I1 for EB F	AAGTTATCGCGGTAAaataaattctttctacacacagtc	overlapped with pT-crtEB R primer
I1 for EB R	CGATAAGCTTGGATCtcacgcctgttcctggctta	overlapped with *Bam*HI site of pT-groG
pTcrtEBI1 F	gatccaagcttatcgatgataa	
pTcrtEBI1 R	tcacgcctgttcctggctta	
Y for EBI1 F	CAGGAACAGGCGTGAggcatggatggtcagtcaat	overlapped with pTcrtEBI1 R primer
Y for EBI1 R	CGATAAGCTTGGATCtcaccccatctcccactcg	overlapped with pTcrtEBI1 F primer
Cfx_cruF F	ATAAGGAGATATACCatgttttccgatcagtctatac	overlapped with *Nco*I site of RSFDuet-1
Cfx_cruF R	GCTCGAATTCGGATCCtcaggggttggtttcggcg	overlapped with *Bam*HI site of RSFDuet-1
Cfx_cruF R2	tcaggggttggtttcggcg	
Cfx_cruC F	GAAACCAACCCCTGAcgaaccatcggatcgttcta	overlapped with Cfx_cruF R2 primer
Cfx_cruC R	GCTCGAATTCGGATCCctaaccggcataccgccg	overlapped with *Bam*HI site of RSFDuet-1
Cfx_cruC R2	ctaaccggcataccgccg	
Cfx_cruD F	CGGTATGCCGGTTAGctgccaacactgacgatgaa	overlapped with Cfx_cruC R2 primer
Cfx_cruD R	GCTCGAATTCGGATCCttatgatcgtgctcgcaggc	overlapped with *Bam*HI site of RSFDuet-1

Table S3. Sequences of primers for the qRT-PCR assays used in this study.

Primer name	Primer sequence
bchE RT-F	gtcccgtgttgcggttatc
bchE RT-R	actggcgcaaggctttatca
crtB RT-F	cgcgatctgcgaacaaatca
crtB RT-R	gtaatcccgatcaccagcaa
crtI1 RT-F	gattcgcctcaaatcgtacc
crtI1 RT-R	gtcagtttgcactttgcggt
crtI2 RT-F	ctgggagagcttatacgatc
crtI2 RT-R	acgactactgcatcagcctt
crtI3 RT-F	atgcgacctacaacggttca
crtI3 RT-R	tcagggatcaggtgagtgta
crtY RT-F	taatagcgtgtatcggccag
crtY RT-R	tcatcgggatgaccccatat
cruF RT-F	ctcatgggattctggttgac
cruF RT-R	atgccgtaatagactccacc
cruC RT-F	aattggccgttgtgtgcaag
cruC RT-R	ggcgctcaaaaggaaagatg
cruD RT-F	atgcaccgtgcgtttgatag
cruD RT-R	gcgccggattgcttcaatat
crtO RT-F	ccaactgatcgaagagacct
crtO RT-R	ccagttgcaggaaagttgtc
idi RT-F	aagattaatctggcgctggc
idi RT-R	atcgataatgcgcacccta
rpoD RT-F	acggcaacaaggcttcgtta
rpoD RT-R	tcttcacccttcgccttcaa

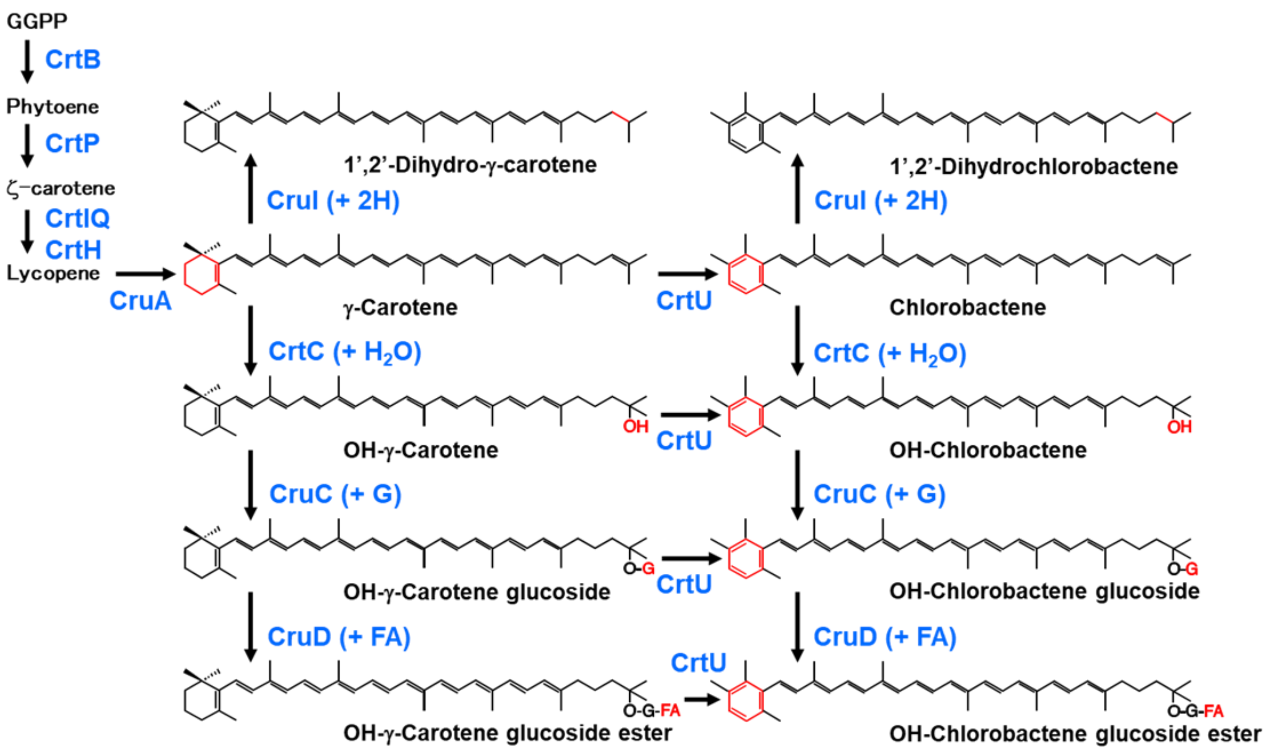

Figure S1. Carotenoid biosynthetic pathway of *Cba. tepidum*. γ-Carotene, chlorobactene, and their derivatives are produced. CrtE is absent. For phytoene to lycopene production, three enzymes of CrtP, CrtQ, and CrtH are used. Lycopene cyclase is CruA-type, not CrtY-type. Abbreviations: FPP, farnesyl pyrophosphate; FA, fatty acid; G, glucoside.

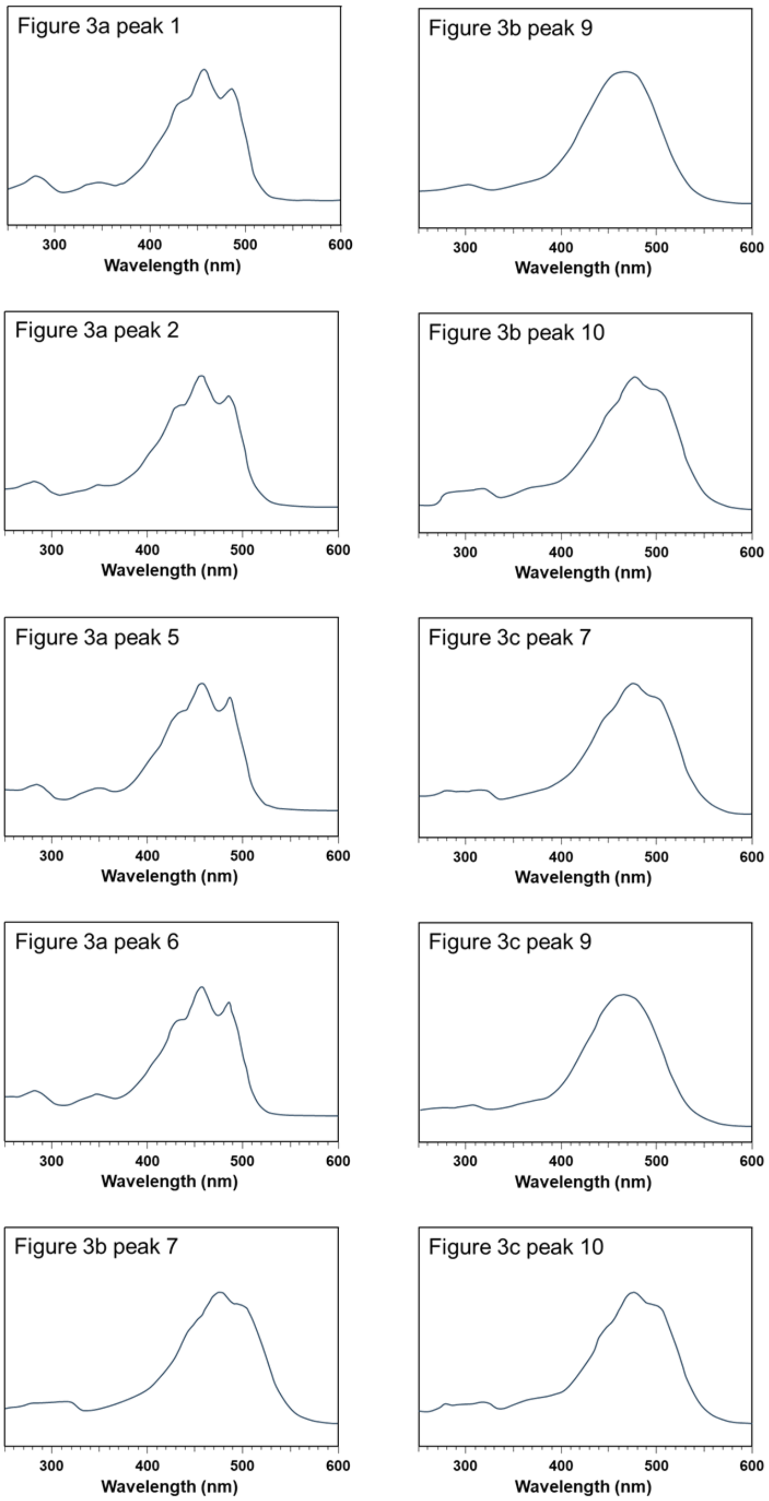

Figure S2. Absorption spectra of major carotenoids from *Cfx. aurantiacus* detected in Figure 3. Peak 1, OH-γ-carotene glucoside; peak2, OH-γ-carotene; peak 5, OH-γ-carotene glucoside ester; peak 6, OH-γ-carotene glucoside ester; peak 7, keto-OH-γ-carotene glucoside; peak 9, canthaxanthin; and peak 10, keto-γ-carotene.

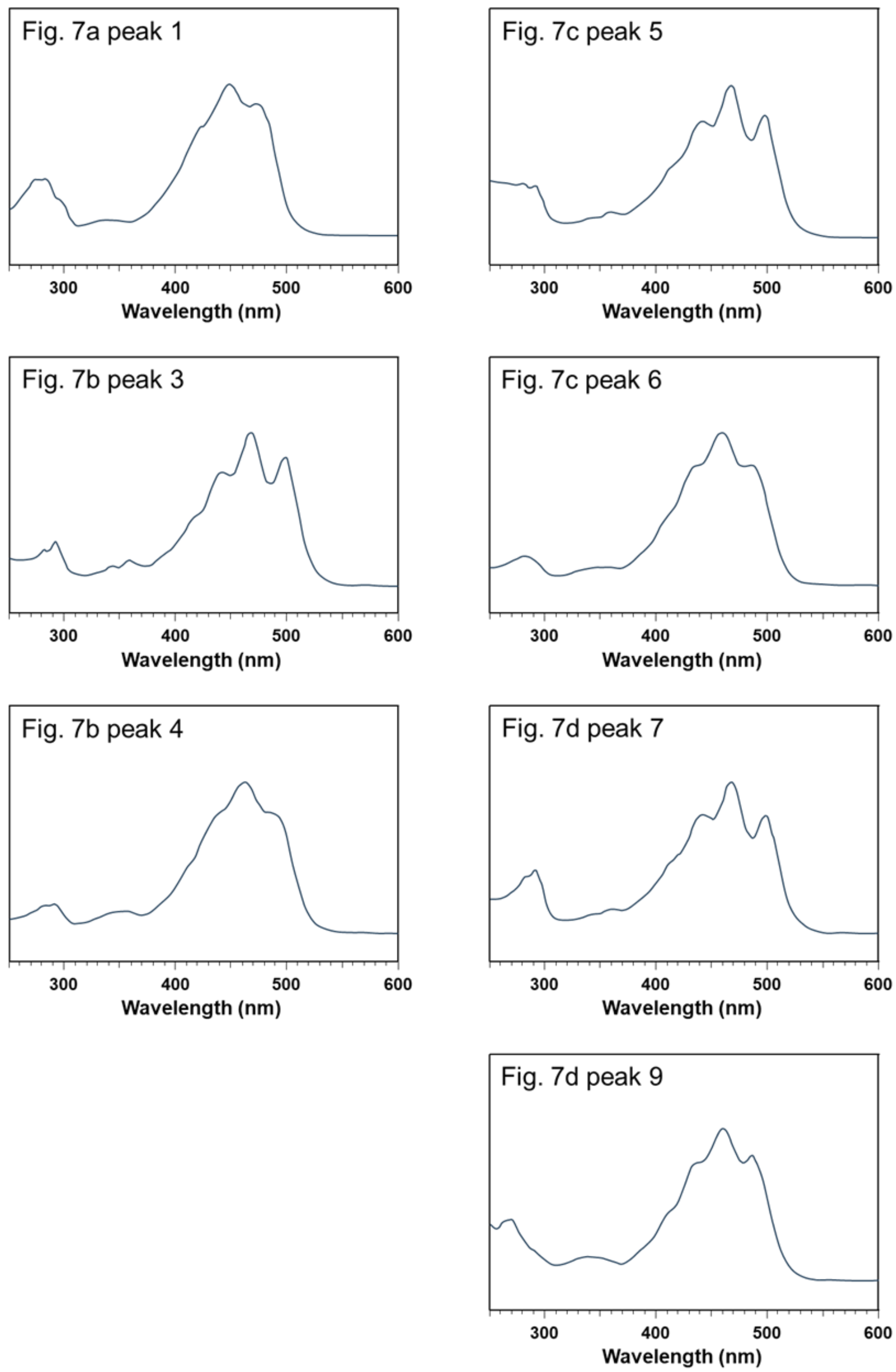

Figure S3. Absorption spectra of major carotenoids detected in Figure 7. Peak 1, phytoene; peak 3, di-OH-lycopene; peak 4, OH-lycopene; peak 5, di-OH-lycopene-di-glucoside; peak 6, OH-γ-carotene glucoside; peak 7, di-OH-lycopene di-glucoside ester; and peak 9, OH-γ-carotene glucoside ester.
